# Alavi–Carlsen Calcification Score (ACCS): A Simple Measure of Global Cardiac Atherosclerosis Burden

**DOI:** 10.3390/diagnostics11081421

**Published:** 2021-08-05

**Authors:** Babak Saboury, Lars Edenbrandt, Reza Piri, Oke Gerke, Tom Werner, Armin Arbab-Zadeh, Abass Alavi, Poul Flemming Høilund-Carlsen

**Affiliations:** 1 Clinical Center, Department of Radiology and Imaging Sciences, National Institutes of Health, Bethesda, MD 20892, USA; babak.saboury@nih.gov; 2 Department of Computer Science and Electrical Engineering, University of Maryland, Baltimore County, Baltimore, MD 21250, USA; 3 Department of Radiology, Hospital of the University of Pennsylvania, Philadelphia, PA 19104, USA; tom.werner@pennmedicine.upenn.edu; 4 Department of Molecular and Clinical Medicine, Institute of Medicine, Sahlgrenska Academy, University of Gothenburg, 41345 Gothenburg, Sweden; lars.edenbrandt@gu.se; 5 Department of Clinical Physiology, Sahlgrenska University Hospital, Region Västra Götaland, 41345 Gothenburg, Sweden; 6 Department of Nuclear Medicine, Odense University Hospital, 5000 Odense C, Denmark; reza.piri2@rsyd.dk (R.P.); oke.gerke@rsyd.dk (O.G.); 7 Department of Clinical Research, University of Southern Denmark, 5000 Odense C, Denmark; 8 Division of Cardiology, Department of Medicine, Johns Hopkins University School of Medicine, Baltimore, MD 21287, USA; azadeh1@jhmi.edu

**Keywords:** aorta, artificial intelligence, atherosclerosis, calcium, heart, imaging, microcalcification, PET/CT, score, sodium fluoride

## Abstract

Multislice cardiac CT characterizes late stage macrocalcification in epicardial arteries as opposed to PET/CT, which mirrors early phase arterial wall changes in epicardial and transmural coronary arteries. With regard to tracer, there has been a shift from using mainly ^18^F-fluorodeoxyglucose (FDG), indicating inflammation, to applying predominantly ^18^F-sodium fluoride (NaF) due to its high affinity for arterial wall microcalcification and more consistent association with cardiovascular risk factors. To make NaF-PET/CT an indispensable adjunct to clinical assessment of cardiac atherosclerosis, the Alavi–Carlsen Calcification Score (ACCS) has been proposed. It constitutes a global assessment of cardiac atherosclerosis burden in the individual patient, supported by an artificial intelligence (AI)-based approach for fast observer-independent segmentation. Common measures for characterizing epicardial coronary atherosclerosis by NaF-PET/CT as the maximum standardized uptake value (SUV) or target-to-background ratio are more versatile, error prone, and less reproducible than the ACCS, which equals the average cardiac SUV. The AI-based approach ensures a quick and easy delineation of the entire heart in 3D to obtain the ACCS expressing ongoing global cardiac atherosclerosis, even before it gives rise to CT-detectable coronary calcification. The quantification of global cardiac atherosclerotic burden by the ACCS is suited for management triage and monitoring of disease progression with and without intervention.

## 1. Historical Background

Three major trends have dominated cardiac atherosclerosis imaging for the last 70 years. After World War II, invasive coronary angiography was developed to guide coronary artery bypass grafting and later percutaneous transluminal intervention. In the 1970s and 1980s, non-invasive myocardial perfusion imaging with ^201^thallium and later ^99m^Tc-labeled tracers was introduced, and this millennium has given rise to multislice cardiac CT, which has reduced perfusion imaging to assessment primarily of hemodynamic consequences of CT-detected coronary stenosis. Additionally, quantification of coronary calcium deposits as a risk factor for future cardiac events was undertaken by electron beam CT in the 1980s, until it was largely replaced by ultra-fast multislice cardiac CT.

In general, these techniques characterize arterial wall macrocalcification, i.e., calcium deposits > 50 µm that occur late in the disease process [1], or its consequences, when curative treatment is no longer an option. Additionally, they cannot detect molecular calcium deposits ≤ 50 µm or their detrimental effects in early stage atherosclerosis, when the disease is still asymptomatic, but presumably more sensitive to intervention than later on when symptoms give rise to diagnostic work-up.

## 2. Molecular Imaging in Atherosclerosis

Therefore, there are compelling reasons to look for methods that can detect early stage atherosclerosis in an easy, rapid, and reliable way. Molecular imaging with ^18^F-sodium fluoride (NaF) PET/CT is an obvious choice. Several tracers have been proposed for imaging atherosclerosis [2], but only two have achieved significant use, ^18^F-fluorodeoxyglucose (FDG) and NaF. FDG was suggested for this purpose twenty years ago [3,4], whereas NaF was not applied until nearly 10 years later [5,6]. FDG was originally introduced for imaging of regional cerebral metabolism in healthy aging and neuro-psychiatric disorders [7,8] while NaF was initially used to demonstrate bone abnormalities [9].

With regard to atherosclerosis, the two tracers mirror arterial wall inflammation and microcalcification, respectively [2]. With time, focus has shifted from FDG towards NaF. Opposite to arterial NaF retention, arterial FDG accumulation does not correlate with CT calcium burden [10,11]. Arterial wall NaF uptake is more consistently related to cardiovascular risk factors than FDG accumulation [11,12,13,14,15], which tends to come and go with time [16], whereas arterial NaF deposits are more consistently present, at least in patients with angina compared to healthy control subjects [17]. Moreover, it has been demonstrated in the Ossabaw swine model of metabolic syndrome that (a) coronary NaF uptake precedes macroscopic calcification on intravascular ultrasound and CT scans and (b) NaF does not accumulate in the myocardium [18], opposite to the high physiologic myocardial uptake of FDG, hampering FDG-PET imaging of coronary atherosclerosis [2,19]. Finally, arterial uptake and blood background clearance of NaF occur much faster than with FDG [12]. Thus, NaF appears to be the more promising PET tracer for imaging cardiac atherosclerosis [2,20].

What remains to make NaF-PET an indispensable adjunct to clinical assessment of atherosclerosis? There are two obstacles preventing clinical implementation of NaF-PET imaging: (1) demonstration in humans of a time-dependent close association between NaF-detectable arterial microcalcification in early stage atherosclerosis and later CT-detectable macrocalcification and (2) rapid and reliable methods providing within minutes a valid assessment of the extent and activity of ongoing cardiac atherosclerosis. The former requires repeat NaF-PET imaging on the same scanner in longitudinal studies, which have still not been conducted [21,22], whereas, for the latter, we propose using artificial intelligence (AI)-based determination of what we have termed the Alavi–Carlsen Calcification Score [18,23,24,25,26].

## 3. Alavi–Carlsen Calcification Score (ACCS)

The ACCS is based on the concept of global disease assessment [23], inspired by a milestone review by Arbab-Zadeh and Fuster [27] arguing for “a transitioning from a focus on individual lesions to atherosclerotic disease burden for coronary artery disease risk assessment”. The concept of global disease assessment was introduced in 1993 for FDG uptake imaging of the brain [28]. It can be used for the entire body, tumor tissue, or specific organs such as the heart [19,29,30], assuming that total tracer uptake, i.e., the weighted average uptake in all diseased lesions, is a truer indicator of the extent and aggressiveness of the disease than simple measures like diameter on an X-ray or the maximal SUV in a single voxel of the affected tissues [23]. The aforementioned review concludes that a state of generalized vulnerability is more important than characterizing the individual sites of vulnerability in the individual patient, since plaque rupture often occurs without clinical symptoms, plaque morphology changes over a few months, and plaque rupture frequently occurs away from the culprit lesions [27]. In addition to coronary plaque burden, the activity of atherosclerosis, assessed by plaque progression over time, is strongly associated with acute coronary event risk [31]. In line with this, the ACCS represents a shift in nuclear cardiology from diagnosing late stage coronary artery stenosis and its consequences to providing a measure of early atherosclerosis burden and its activity in the entire coronary arterial tree, including transmural and minor arteries subtending the most ischemia-prone parts of the myocardium.

**Figure 1:**
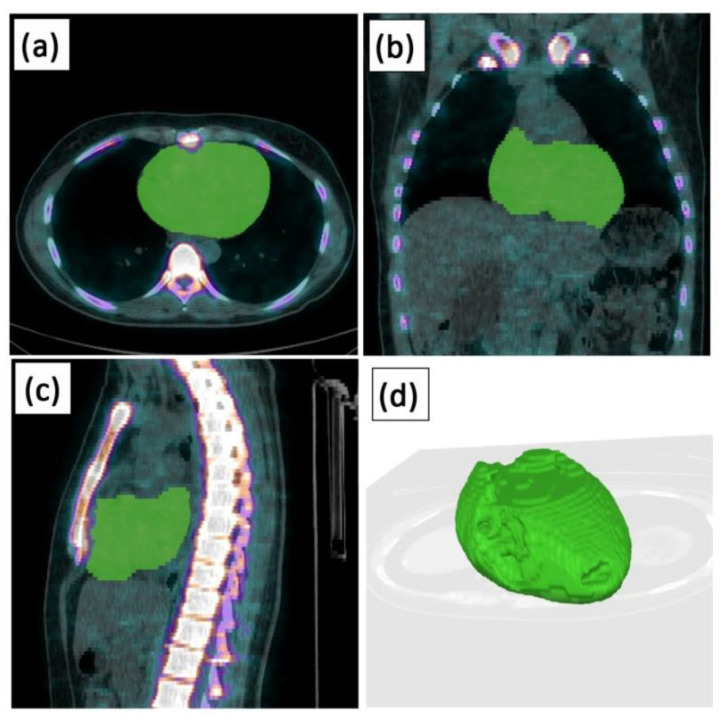
Axial (**a**), coronal (**b**), and sagittal (**c**) reconstruction of AI-based cardiac segmentation in the same patient and 2D representation of the 3D reconstructed heart using AI (**d**).

The ACCS represents early phase global cardiac atherosclerosis burden rather than characterizing late-phase atherosclerotic calcification in vulnerable coronary plaques in proximal coronary arteries by measures like the target-to-background ratio (TBR) [32,33,34,35,36,37]. In contrast, the ACCS is the mean standardized uptake NaF value within the entire cardiac silhouette in 3D, i.e., average cardiac standardized uptake value (SUV), excluding the aortic offspring of the coronary arteries and including cardiac blood pool activity (Figure 1), which is why we have chosen the designation “cardiac” instead of “coronary” calcification burden.

The ACCS is equal to the average cardiac SUVmean and can be measured by segmenting the entire heart from the surrounding tissues to obtain the average cardiac SUV. Manual segmentation typically lasts half an hour per patient and is associated with some variability, whereas the AI-based approach yields the same measure in less than a minute and with a reproducibility of 100% at reanalysis of the same PET/CT scan. The principle of the AI-based method is organ segmentation using multiple labels in the CT images in two so-called convoluted neural networks trained beforehand in a separate set of scans. One of these networks handles bone segmentation and the other deals with other, non-bone labels, as described in detail elsewhere [38]. The main challenge with both approaches when it comes to heart segmentation is the cranial delineation of the heart in non-contrast CT scans [39]. However, with continued learning, it is foreseeable that this and other challenges will diminish and almost disappear. An inevitable limitation is the inclusion of cardiac blood activity, as delineation of the endocardial surface is impossible with manual segmentation and not accounted for with AI-based segmentation to date due to the complicated structure of the trabeculae carneae. However, with the rapid blood clearance of NaF [40], this is a minor problem if the same acquisition time following tracer administration is used at repeat scanning.

## 4. Current Measures of Late Stage Coronary Atherosclerosis

Present common semi-automated measures of late stage coronary atherosclerosis are the Agatston score obtained by non-contrast CT and the SUVmax and the TBR calculated from NaF-PET/CT scans. The Agatston score is a useful tool to calculate coronary artery calcification from a low-dose CT scan. It is the product of the maximum attenuation value in Hounsfield units (HU) multiplied by the area of a calcification [41,42]. It has an arbitrary lower cut-off of typically 130 HU, meaning that early state calcification on the molecular level is not considered. Being a fairly reproducible measure, it allows for long-term monitoring of coronary calcification development, and is sensitive enough to record an unexpected 2-year decrease in coronary calcification in healthy control subjects in concert with a similarly unexpected 2-year decrease in global NaF uptake [17].

Common measures of NaF uptake, such as the SUVmax or TBR, are more versatile, error prone, and less reproducible. Nonetheless, SUVmax appears in a multitude of PET studies of many diseases, although it is, in principle, an extreme stand-alone outlier, which cannot serve as reliable indicator of the extent and activity of disease. When it comes to coronary uptake of PET tracers, there is no standardization. Thus, in 2015, when reviewing 49 articles, Huet et al. [43] counted 53 different acquisition protocols, 51 reconstruction protocols, and 46 quantification methods to characterize atherosclerotic lesions on FDG PET scans. The picture has hardly changed since then, and conditions are no different with respect to quantifying NaF uptake. The often-applied TBR is an error-prone quantity, not only because coronary plaques are difficult to detect and delineate due to movements of the heart and lungs, but also because of the limited spatial resolution of PET, which requires partial volume correction to adjust for count loss in very small lesions [44,45]. Moreover, calculated TBR is strongly dependent on registered blood background activity, which is a major confounder with NaF because it varies from one vessel section to another due to cross-talk from the high uptake in adjacent bone [46]. It is particularly unfortunate that TBR increases with time from tracer administration to image acquisition [39,46,47]. Finally, all sources of error come into play twice with repeat measurements [48], rendering TBR suboptimal for monitoring changes in coronary NaF uptake.

Most of the sources of error are of minor importance when measuring the ACCS. With the AI-based approach, it is quick and easy to delineate the entire heart in 3D to obtain the ACCS expressing ongoing global cardiac atherosclerosis, even before it gives rise to CT-detectable coronary calcification. The large volume of the heart renders partial volume correction superfluous, and inclusion of cardiac blood pool activity is no problem. Given the good reproducibility of the AI method, this approach is particularly suited for characterizing individual patients and following changes over time.

## 5. Conclusions

Traditional imaging of coronary atherosclerosis and its hemodynamic consequences depicts late stage atherosclerotic changes in epicardial coronary arteries and their consequences for myocardial perfusion. Instead, AI-based assessment of global cardiac NaF uptake in the form of the ACCS offers fast and reliable assessment of global cardiac atherosclerotic burden suited for management triage and monitoring of disease progression with and without intervention.

## References

[1] Kelly-Arnold A., Maldonado N., Laudier D., Aikawa E., Cardoso L., Weinbaum S. (2013). Revised microcalcification hypothesis for fibrous cap rupture in human coronary arteries. Proc. Natl. Acad. Sci. USA.

[2] Høilund-Carlsen P.F., Moghbel M., Gerke O., Alavi A. (2019). Evolving role of PET in detecting and characterizing atherosclerosis. PET Clin..

[3] Yun M., Yeh D., Araujo L.I., Jang S., Newberg A., Alavi A. (2001). F-18 FDG uptake in the large arteries: A new observation. Clin. Nucl. Med..

[4] Yun M., Jang S., Cucchiara A., Newberg A.B., Alavi A. (2002). 18F FDG uptake in the large arteries: A correlation study with the atherogenic risk factors. Semin. Nucl. Med..

[5] Derlin T., Richter U., Bannas P., Begemann P., Buchert R., Mester J., Klutmann S. (2010). Feasibility of 18F-sodium fluoride PET/CT for imaging of atherosclerotic plaque. J. Nucl. Med..

[6] Derlin T., Wisotzki C., Richter U., Apostolova I., Bannas P., Weber C., Mester J., Klutmann S. (2011). In Vivo imaging of mineral deposition in carotid plaque using 18F-sodium fluoride PET/CT: Correlation with atherogenic risk factors. J. Nucl. Med..

[7] Alavi A., Reivich M. (2002). Guest editorial: The conception of FDG-PET imaging. Semin. Nucl. Med..

[8] Newberg A., Alavi A., Reivich M. (2002). Determination of regional cerebral function with FDG-PET imaging in neuropsychiatric disorders. Semin. Nucl. Med..

[9] Blau M., Nagler W., Bender M.A. (1962). Fluorine-18: A new isotope for bone scanning. J. Nucl. Med..

[10] Derlin T., Tóth Z., Papp L., Wisotzki C., Apostolova I., Habermann C.R., Mester J., Klutmann S. (2011). Correlation of inflammation assessed by 18F-FDG PET, active mineral deposition assessed by 18F-fluoride PET, and vascular calcification in atherosclerotic plaque: A dual-tracer PET/CT study. J. Nucl. Med..

[11] Dweck M.R., Chow M.W., Joshi N.V., Williams M.C., Jones C., Fletcher A.M., Richardson H., White A., McKillop G., van Beek E.J. (2012). Coronary arterial 18F-sodium fluoride uptake: A novel marker of plaque biology. J. Am. Coll. Cardiol..

[12] Blomberg B.A., de Jong P.A., Thomassen A., Lam M.G.E., Vach W., Olsen M.H., Mali W.P.T.M., Narula J., Alavi A., Høilund-Carlsen P.F. (2017). Thoracic aorta calcification but not inflammation is associated with increased cardiovascular disease risk: Results of the CAMONA study. Eur. J. Nucl. Med. Mol. Imaging.

[13] Arani L.S., Gharavi M.H., Zadeh M.Z., Raynor W., Seraj S.M., Constantinescu C.M., Gerke O., Werner T.J., Høilund-Carlsen P.F., Alavi A. (2019). Association between age, uptake of 18F-fluorodeoxyglucose and of 18F-sodium fluoride, as cardiovascular risk factors in the abdominal aorta. Hell. J. Nucl. Med..

[14] Sorci O., Batzdorf A.S., Mayer M., Rhodes S., Peng M., Jankelovits A.R., Hornyak J.N., Gerke O., Høilund-Carlsen P., Alavi A. (2020). 18F-sodium fluoride PET/CT provides prognostic clarity compared to calcium and Framingham risk scoring when addressing whole-heart arterial calcification. Eur. J. Nucl. Med. Mol. Imaging.

[15] Morbelli S., Fiz F., Piccardo A., Picori L., Massollo M., Pestarino E., Marini C., Cabria M., Democrito A., Cittadini G. (2014). Divergent determinants of 18F–NaF uptake and visible calcium deposition in large arteries: Relationship with Framingham risk score. Int. J. Cardiovasc. Imaging.

[16] Meirelles G.S., Gonen M., Strauss H.W. (2011). 18F-FDG uptake and calcifications in thoracic aorta on positron emission tomography/computed tomography examinations: Frequency and stability of serial scans. J. Thorac. Imaging.

[17] Piri R., Lici G., Riyahimanesh P., Gerke O., Alavi A., Høilund-Carlsen P.F. (2021). Two-year change in 18F-sodium fluoride uptake in major arteries of healthy subjects and angina pectoris patients. Int. J. Cardiovasc. Imaging.

[18] McKenney-Drake M.L., Moghbel M.C., Paydary K., Alloosh M., Houshmand S., Moe S., Salavati A., Sturek J.M., Territo P.R., Weaver C. (2018). 18F-NaF and 18F-FDG as molecular probes in the evaluation of atherosclerosis. Eur. J. Nucl. Med. Mol. Imaging.

[19] Alavi A., Werner T.J., Høilund-Carlsen P.F. (2018). What can be and what cannot be accomplished with PET to detect and characterize atherosclerotic plaques. J. Nucl. Cardiol..

[20] Moghbel M., Al-Zaghal A., Werner T.J., Constantinescu C.M., Høilund-Carlsen P.F., Alavi A. (2018). The Role of PET in Evaluating Atherosclerosis: A Critical Review. Semin. Nucl. Med..

[21] Høilund-Carlsen P.F., Sturek M., Alavi A., Gerke O. (2020). Atherosclerosis imaging with 18F-sodium fluoride PET: State-of-the-art review. Eur. J. Nucl. Med. Mol. Imaging.

[22] Høilund-Carlsen P.F., Piri R., Constantinescu C., Iversen K.K., Sturek M., Werner T.J., Alavi A., Gerke O. (2020). Atherosclerosis Imaging with 18F-Sodium Fluoride PET. Diagnostics.

[23] Høilund-Carlsen P.F., Edenbrandt L., Alavi A. (2019). Global disease score (GDS) is the name of the game!. Eur. J. Nucl. Med. Mol. Imaging.

[24] Gonuguntla K., Rojulpote C., Patil S., Bhattaru A., Karambelkar P., Vuthaluru K., Raynor W.Y., Borja A.J., Zhang V., Werner T.J. (2020). Utilization of NaF-PET/CT in assessing global cardiovascular calcification using CHADS2 and CHADS2-VASc scoring systems in high risk individuals for cardiovascular disease. Am. J. Nucl. Med. Mol. Imaging.

[25] Borja A.J., Bhattaru A., Rojulpote C., Hancin E.C., Detchou D.K., Patil S., Gonuguntla K., Karambelkar P., Chinta S., Vuthaluru K. (2020). Association between atherosclerotic cardiovascular disease risk score estimated by pooled cohort equation and coronary plaque burden as assessed by NaF-PET/CT. Am. J. Nucl. Med. Mol. Imaging.

[26] Paydary K., Revheim M.E., Emamzadehfard S., Gholami S., Pourhassan S., Werner T.J., Høilund-Carlsen P.F., Alavi A. (2021). Quantitative thoracic aorta calcification assessment by 18F-NaF PET/CT and its correlation with atherosclerotic cardiovascular disorders and increasing age. Eur. Radiol..

[27] Arbab-Zadeh A., Fuster V. (2015). The myth of the "vulnerable plaque": Transitioning from a focus on individual lesions to atherosclerotic disease burden for coronary artery disease risk assessment. J. Am. Coll. Cardiol..

[28] Alavi A., Newberg A.B., Souder E., Berlin J.A. (1993). Quantitative analysis of PET and MRI data in normal aging and Alzheimer’s disease: Atrophy weighted total brain metabolism and absolute whole brain metabolism as reliable discriminators. J. Nucl. Med..

[29] Basu S., Zaidi H., Salavati A., Hess S., Carlsen P.F., Alavi A. (2014). FDG PET/CT methodology for evaluation of treatment response in lymphoma: From “graded visual analysis” and “semiquantitative SUVmax” to global disease burden assessment. Eur. J. Nucl. Med. Mol. Imaging.

[30] Alavi A., Werner T.J., Høilund-Carlsen P.F. (2017). What can be and what cannot be accomplished with PET: Rectifying ongoing misconceptions. Clin. Nucl. Med..

[31] Arbab-Zadeh A. (2021). The PROMISE and challenges of whole-heart atherosclerosis imaging. J. Cardiovasc. Comput. Tomogr..

[32] Adamson P.D., Vesey A.T., Joshi N.V., Newby D.E., Dweck M.R. (2015). Salt in the wound: (18)F-fluoride positron emission tomography for identification of vulnerable coronary plaques. Cardiovasc. Diagn. Ther..

[33] Lee J.M., Bang J.I., Koo B.K., Hwang D., Park J., Zhang J., Yaliang T., Suh M., Paeng J.C., Shiono Y. (2017). Clinical Relevance of 18F-Sodium Fluoride Positron-Emission Tomography in Noninvasive Identification of High-Risk Plaque in Patients with Coronary Artery Disease. Circ. Cardiovasc. Imaging.

[34] Kitagawa T., Yamamoto H., Nakamoto Y., Sasaki K., Toshimitsu S., Tatsugami F., Awai K., Hirokawa Y., Kihara Y. (2018). Predictive Value of (18)F-Sodium Fluoride Positron Emission Tomography in Detecting High-Risk Coronary Artery Disease in Combination with Computed Tomography. J. Am. Heart Assoc..

[35] Marchesseau S., Seneviratna A., Sjöholm A.T., Qin D.L., Ho J.X.M., Hausenloy D.J., Townsend D.W., Richards A.M., Totman J.J., Chan M.Y.Y. (2018). Hybrid PET/CT and PET/MRI imaging of vulnerable coronary plaque and myocardial scar tissue in acute myocardial infarction. J. Nucl. Cardiol..

[36] Hop H., de Boer S.A., Reijrink M., Kamphuisen P.W., de Borst M.H., Pol R.A., Zeebregts C.J., Hillebrands J.L., Slart R.H.J.A., Boersma H.H. (2018). 18F-sodium fluoride positron emission tomography assessed microcalcifications in culprit and non-culprit human carotid plaques. J. Nucl. Cardiol..

[37] Joshi N.V., Vesey A.T., Williams M.C., Shah A.S., Calvert P.A., Craighead F.H., Yeoh S.E., Wallace W., Salter D., Fletcher A.M. (2014). 18F-fluoride positron emission tomography for identification of ruptured and high-risk coronary atherosclerotic plaques: A prospective clinical trial. Lancet.

[38] Trägårdh E., Borrelli P., Kaboteh R., Gillberg T., Ulén J., Enqvist O., Edenbrandt L. (2020). RECOMIA—A cloud-based platform for artificial intelligence research in nuclear medicine and radiology. EJNMMI Phys..

[39] Piri R., Edenbrandt L., Larsson M., Enqvist O., Skovrup S., Iversen K.K., Alavi A., Gerke O., Høilund-Carlsen P.F. (2021). Global cardiac atherosclerotic burden assessed by artificial intelligence-based versus manual segmentation in 18F-sodium fluoride PET/CT scans: Head-to-head comparison. J. Nucl. Cardiol..

[40] Blomberg B.A., Thomassen A., Takx R.A., Vilstrup M.H., Hess S., Nielsen A.L., Diederichsen A.C., Mickley H., Alavi A., Høilund-Carlsen P.F. (2014). Delayed sodium 18F-fluoride PET/CT imaging does not improve quantification of vascular calcification metabolism: Results from the CAMONA study. J. Nucl. Cardiol..

[41] Agatston A.S., Janowitz W.R., Hildner F.J., Zusmer N.R., Viamonte M., Detrano R. (1990). Quantification of coronary artery calcium using ultrafast computed tomography. J. Am. Coll. Cardiol..

[42] Greenland P., Alpert J.S., Beller G.A., Benjamin E.J., Budoff M.J., Fayad Z.A., Foster E., Hlatky M.A., Hodgson J.M., Kushner F.G. (2010). American College of Cardiology Foundation; American Heart Association. 2010 ACCF/AHA guideline for assessment of cardiovascular risk in asymptomatic adults: A report of the American College of Cardiology Foundation/American Heart Association Task Force on Practice Guidelines. J. Am. Coll. Cardiol..

[43] Huet P., Burg S., Le Guludec D., Hyafil F., Buvat I. (2015). Variability and uncertainty of 18F-FDG PET imaging protocols for assessing inflammation in atherosclerosis: Suggestions for improvement. J. Nucl. Med..

[44] Chen W., Dilsizian V. (2015). PET assessment of vascular inflammation and atherosclerotic plaques: SUV or TBR?. J. Nucl. Med..

[45] Alavi A., Werner T.J., Høilund-Carlsen P.F., Zaidi H. (2018). Correction for Partial Volume Effect Is a Must, Not a Luxury, to Fully Exploit the Potential of Quantitative PET Imaging in Clinical Oncology. Mol. Imaging Biol..

[46] Blomberg B.A., Thomassen A., de Jong P.A., Simonsen J.A., Lam M.G., Nielsen A.L., Mickley H., Mali W.P., Alavi A., Høilund-Carlsen P.F. (2015). Impact of Personal Characteristics and Technical Factors on Quantification of Sodium 18F-Fluoride Uptake in Human Arteries: Prospective Evaluation of Healthy Subjects. J. Nucl. Med..

[47] Blomberg B.A., Akers S.R., Saboury B., Mehta N.N., Cheng G., Torigian D.A., Lim E., Del Bello C., Werner T.J., Alavi A. (2013). Delayed time-point 18F-FDG PET CT imaging enhances assessment of atherosclerotic plaque inflammation. Nucl. Med. Commun..

[48] Høilund-Carlsen P.F., Lauritzen S.L., Marving J., Rasmussen S., Hesse B., Folke K., Godtfredsen J., Chraemmer-Jørgensen B., Gadsbøll N., Dige-Petersen H. (1988). The reliability of measuring left ventricular ejection fraction by radionuclide cardiography: Evaluation by the method of variance components. Br. Heart J..

